# Effects of different vaccination regimes on the immunodiagnosis of tuberculosis in goats and evaluation of defined antigens

**DOI:** 10.3389/fvets.2024.1524461

**Published:** 2025-01-15

**Authors:** Patricia Cuenca-Lara, Miriam Blay-Benach, Zoraida Cervera, Cristian Melgarejo, Julia Moraleda, Iker A. Sevilla, Joseba M. Garrido, Mahavir Singh, Gareth J. Jones, Bernat Pérez de Val

**Affiliations:** ^1^Unitat Mixta d’Investigació IRTA-UAB en Sanitat Animal, Centre de Recerca en Sanitat Animal, Campus de la Universitat Autònoma de Barcelona, Bellaterra, Spain; ^2^IRTA, Animal Health, Centre de Recerca en Sanitat Animal, Campus de la Universitat Autònoma de Barcelona, Bellaterra, Spain; ^3^Animal Health Department, NEIKER-Instituto Vasco de Investigación y Desarrollo Agrario, Basque Research and Technology Alliance, Derio, Spain; ^4^Lionex Diagnostics and Therapeutics GmbH, Braunschweig, Germany; ^5^Department of Bacteriology, Animal and Plant Health Agency, Addlestone, United Kingdom

**Keywords:** vaccines, *Mycobacterium bovis*, tuberculosis, diagnosis, skin test, IGRA, DIVA

## Abstract

Tuberculosis (TB) in goats is a chronic infectious disease caused by *Mycobacterium tuberculosis* complex (MTBC) organisms that pose a great health and economic challenge for the caprine industry in some European and developing countries. It is also a zoonotic disease posing a risk for public health. The control programs of the disease are based on a test-and-slaughter strategy, and vaccination is not feasible with available vaccines due to its interferences with the current TB immunodiagnosis. There is still a need for the development of an effective TB vaccine and, concurrently, diagnostic methods that allow differentiation between infected and vaccinated animals (DIVA approach). In this study, we investigated the interferences caused by the tuberculin (PPD)-based TB diagnostic tests in goats immunized by different mucosal and parenteral vaccination strategies: three single-dose strategies based on intranasal administration of BCG and two heat-inactivated *M. bovis* (HIMB) vaccines, and two prime-boost strategies based on parenteral BCG or HIMB priming and intranasal HIMB boosting. In addition, the defined antigens ESAT-6, CPF10, and EspC were evaluated as alternative diagnostic reagents to PPDs. At week 14 after prime vaccination of the animals, skin tests, IFN-*γ* release assay, and antibody detection assays were performed. The two prime-boosted and the single-dose intranasal BCG groups displayed greater cell-mediated immune responses to PPDs than the two single-dose intranasal HIMB vaccines. However, the use of reagents based on the defined antigens eliminated or reduced the vaccine-induced diagnostic interferences in all groups. Based on these results, the use of defined antigens in the current immunodiagnostic tests appears to be suitable in a future goat TB vaccination scenario.

## 1 Introduction

Mammalian TB is a chronic infectious disease of animals and humans that results from infection with members of the *Mycobacterium tuberculosis complex* (MTBC) (1). The two main species of the MTBC affecting domestic small ruminants are *M. caprae* and *M. bovis* (2). TB in goats represents a great health challenge for the caprine industry in Spain and can pose a risk of new TB outbreaks in cattle. Spain ranks second in the European Union for goat population and is also the second-largest producer of goat milk and milk products (3). In spite of this, TB in goats is not subjected to official eradication campaigns; however, control programs applied in bovine tuberculosis control can be adapted in caprine herds (4). The control strategy used at present is based on the test and slaughter of positive animals. The primary screening tests in animals are the single intradermal tuberculin (SIT) test and the comparative intradermal tuberculin (CIT) test, and the *in vitro* IFN-*γ* release assay (IGRA) as ancillary method (5). These diagnostic techniques measure the cell-mediated immune (CMI) response to *M. bovis* and *M. avium* tuberculins (PPDB and PPDA, respectively) (6).

Nowadays, the only available vaccine against mycobacterial infection is Bacille Calmette-Guerin (BCG), a live attenuated strain of *M. bovis*, which confers variable protection in vaccinated animals (7). An additional challenge to the use of BCG or other whole-cell mycobacterial vaccines is that, administered parenterally, they sensitize individuals to respond to the tuberculin-based diagnostic tests, currently used in the TB eradication programs (8, 9). Thus, when studying new vaccination strategies for the control of TB, it is essential for the development and assessment of new diagnostic reagents to distinguish between vaccinated and infected individuals (DIVA approach), enabling the compatibility of the test-and-slaughter strategy with the implementation of vaccination.

In the last two decades, research has focused on identifying new antigens that can serve as DIVA reagents to differentiate between animals that are infected with MTBC and those that have been vaccinated with BCG. Early secretory antigenic target-6 kDa (ESAT-6), culture filtrate protein 10 (CFP10) (10), and, to a lesser extent, ESX-1 secretion-associated protein C (EspC or Rv3615c) (11) are the most promising DIVA reagent candidates (12–14). These antigens have already been tested in regular *antemortem* diagnostic methods (skin test and IGRA) in cattle and goats (15–17).

Most recently, a heat-inactivated *M. bovis* (HIMB) vaccine against TB has also been developed, showing comparable efficacy to BCG when administrated parentally (18, 19). However, HIMB displayed variable degrees of diagnostic interference in parentally vaccinated cattle or goats when using DIVA reagents developed for BCG, ranging from null or low reactivity to skin test in cattle (14) or IGRA in goats (9, 19), to significant interference with IGRA in cattle (20). On the contrary, the oral administration of HIMB can avoid these interferences on TB diagnosis in cattle (20) and goats (21). The effects on the diagnosis after the administration of vaccines through respiratory mucosal routes have not yet been evaluated.

The aim of this study was to assess the effects of different vaccination regimes based on intranasal and parenteral administration of BCG and HIMB vaccines on the tuberculin-based immunodiagnosis of TB in goats. In addition, the performance of the defined antigens ESAT-6, CFP1, and EspC, as an alternative to tuberculins for skin testing and IGRA in these vaccination settings, was also evaluated.

## 2 Materials and methods

### 2.1 Animals and study design

**Figure 1:**
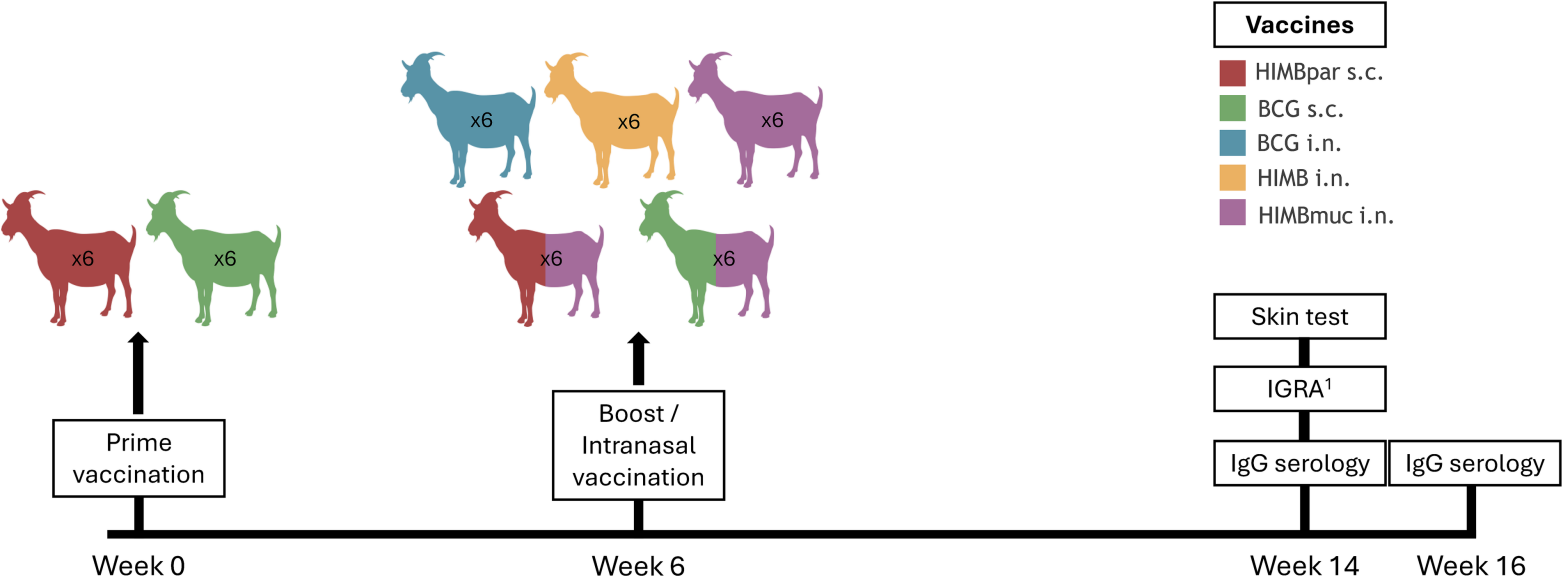
Study design. Goat kids were divided into five groups: (A) *M. bovis* Bacillus Calmette-Guerin (BCG, i.n.); (B) heat-inactivated *M. bovis* (HIMB, i.n.); (C) HIMB with mucosal adjuvant (HIMBmuc, i.n.); (D) homologous prime-boost regimen: priming with HIMB (s.c.) with parenteral adjuvant (HIMBpar) and boosting 6 weeks later with HIMBmuc (i.n.); (E) heterologous prime-boost regimen: priming with BCG (s.c.) and boosting 6 weeks later with HIMBmuc (i.n.). Priming immunizations were carried out at week 0, whereas boosting and single-dose vaccinations were carried out at week 6. Skin tests and IGRAs were performed in all goats at week 14 of the experiment. MTBC-specific antibody response was analyzed at weeks 14 and 16 post-vaccination. ^1^Whole-blood IFNγ release assay.

Thirty Murciano-Granadina goat kids (15 males and 15 females) of approximately 4 months old were randomly divided into 5 groups of 6 animals each (3 males and 3 females): (A) *M. bovis* Bacillus Calmette-Guerin (BCG, i.n.); (B) heat-inactivated *M. bovis* (HIMB, i.n.); (C) HIMB with mucosal adjuvant (HIMBmuc, i.n.); (D) homologous prime-boost regimen: priming with HIMB (s.c.) with parenteral adjuvant (HIMBpar) and boosting 6 weeks later with HIMBmuc (i.n.); (E) heterologous prime-boost regimen: priming with BCG (s.c.) and boosting 6 weeks later with HIMBmuc (i.n.). Two goats (one male and one female) were used as non-vaccinated (NV) controls. Priming immunizations were carried out at week 0, whereas boosting and single-dose vaccinations were carried out at week 6. Figure 1 summarizes the study design.

All animals were confirmed negative for TB by the IFN-*γ* release assay (IGRA, ID Screen® Ruminant IFNg, ID, Grabels, France) before the experiment.

Experimental animals were allocated at Servei de Granges i Camps Experimentals (SGCE) at the Autonomous University of Barcelona (Register No. B9900042). Males and females were separated into two different pens, with 16 animals each. Animals were daily followed up for clinical signs, and body weights were measured every 2 weeks throughout the study. Rectal temperatures were recorded before and at 6, 24, and 48 h after i.n. vaccinations. All animals were bled (10–20 mL) through the jugular vein at weeks 14 and 16. At week 16, all animals were humanely euthanized by intravenous administration of pentobarbital (200 mg/kg).

All experimental procedures were approved by the Animal Welfare Committees of the Autonomous University of Barcelona (Procedure No. 5482-CEEAH-UAB) and the Generalitat de Catalunya (Reference No. 12164). These procedures agreed with the European Union laws for the protection of experimental animals.

### 2.2 Vaccines and vaccination procedure

#### 2.2.1 Bacille Calmette-Guerin

The *M. bovis* BCG Danish strain 1331 (ATCC35733) was prepared as described previously (19). For the subcutaneous vaccination, BCG was diluted in phosphate-buffered saline (PBS) to reach a suspension of ⁓ 10^5^ colony forming units (CFU)/mL, and 1 mL of the suspension was injected subcutaneously into the right scapula.

For the intranasal administration, 1 mL of the suspension of BCG at 2–3·10^7^ CFU/mL was administered intranasally into the right and left nostrils (0.5 mL each).

#### 2.2.2 Heat-inactivated *Mycobacterium bovis* vaccines

HIMB was produced by NEIKER (Derio, Bizkaia, Spain) as previously described (18). HIMB i.n. was administered without adjuvant. HIMBmuc i.n. was adjuvanted with 20% MontanideTM GEL 02 PR (Seppic, Paris, France). All intranasally delivered vaccines were administered as described above for BCG i.n. HIMBpar was adjuvanted with MontanideTM ISA 61 VG (Seppic) and administered as BCG s.c (1 mL injected subcutaneously into the right scapula).

### 2.3 Antigens and peptides

*Mycobacterium bovis* and *M. avium* purified protein derivatives (PPDB and PPDA, respectively, 25,000 IU/mL) were obtained from CZ Vaccines (O Porriño, Pontevedra, Spain). The antigenic cocktail composed of ESAT-6, CFP10, and EspC (Rv3615c) recombinant proteins (DIGRA-C) was formulated as a 1:1:1 mixture at 500 μg/mL each and was obtained from Lionex (Braunschweig, Germany). The ESAT-6-CFP10-EspC recombinant fusion protein (DST-F, 300 μg/mL) (14) and the MTBC-specific antigen MPB83 were also produced by Lionex.

### 2.4 Skin test

Skin tests were performed in all goats at week 14 of the experiment (at 14 or 8 weeks after vaccination or boosting depending on the group) by inoculating intradermally 0.1 mL of PPDB and PPDA into the upper and lower right side of the neck, respectively, and 0.1 mL of DST-F into the left side of the neck, using a Groteerman® syringe (Inserbo S.L., Lleida, Spain). The skin fold thickness was measured before PPDs and DST-F injections and again after 72 h. Skin tests were done following the standard protocols defined by the European Union Reference Laboratory for Bovine TB (VISAVET, Madrid, Spain). As appropriate cutoffs for DST-F have not been evaluated in goats, the interpretation of the PPD-based assay results was done following the indications of VISAVET and adapted to the interpretation of the DST-F-based assays. SIT and DST-F skin tests were considered positive if ∆ skin fold thickness (measures at 72 h minus 0 h) to PPDB or DST-*F* > 2 mm (low cutoff value) or ≥ 4 mm (high cutoff value). CIT was considered positive if positive SIT and PPDB reaction minus PPDA reaction ≥1 mm (low cutoff value) or > 4 mm (high cutoff value).

### 2.5 Whole-blood IFN-*γ* release assay

The IGRAs were performed by collecting whole-blood samples from the jugular vein in heparinized blood tubes from the animals at week 14 post-vaccination. Two aliquots of 1 mL each of whole blood were stimulated in 2.2 mL 96-well cell culture plates (Eppendorf, Hamburg, Germany) with PPDB and PPDA at final concentrations of 20 μg/mL each, and another aliquot of 225 μL of whole blood was stimulated in 300 μL 96-well cell culture plates (Thermo Fisher Scientific, Waltham, MA, United States) with DIGRA-C at a final concentration of 30 μg/mL (10 μg/mL of each antigen—ESAT-6, CFP10, and EspC). PBS was added to cultures as a non-stimulated control. Blood samples were incubated overnight at 37°C with 5% CO2, and plasma supernatants were collected after centrifugation at 1260 g for 10 min and stored at −20°C until further analysis. Plasma samples were defrosted just before performing the IFN-*γ* enzyme-linked immunosorbent assay (ELISA) using in parallel the ID Screen® Ruminant IFNg (ID, Grabels, France) and BOVIGAM TB® (Thermo Fisher Scientific) kits. ELISAs were performed according to the manufacturer’s instructions. ELISA results were obtained as optical density (OD) determined at 450 nm using a spectrophotometer (Biotek Power Wave XS®, Agilent, Santa Clara, United States). In the BOVIGAM TB assay, the ΔOD was calculated as PPDB OD or DIGRA-C OD - PBS OD. A sample was considered positive when ΔOD ≥ 0.1 and PPDB OD > PPDA OD for the tuberculin-based test and ΔOD ≥ 0.1 for the DIGRA-C-based assay. In the ID Screen® Ruminant IFNg assay, the S/P (%) ratio was calculated by dividing (PPDB OD – PPDA OD) or (DIGRA-C OD – PBS OD) by (plate positive control mean OD – plate negative control mean OD) x 100. A sample was classified as positive when S/*p* ≥ 16%. The interpretation of the results of the tuberculin-based assays was done following the indications of the Spanish National Reference Laboratory for bovine TB (LCSA, Santa Fe, Granada, Spain), and an equivalent criterion was adapted for the results interpretation of the DIGRA-C-based assay.

### 2.6 Antibody detection assay

Plasma samples from all experimental animals were analyzed in duplicate to follow-up the MTBC-specific antibody response at weeks 14 and 16 post-vaccination (before and at 2 weeks after skin testing, respectively). An indirect ELISA was used to detect total IgG against MPB83 antigen as described previously (22), determined at 450 nm. MPB83–IgG levels were calculated as the mean OD of antigen-coated well—OD of non-coated well (ΔOD). A sample was classified as positive when ΔOD ≥ 0.5.

### 2.7 Data analysis

Intragroup comparisons between tuberculin-based and defined antigen-based tests were performed within each treatment group. SIT, CIT, and DST-F results, measured as skin thickness increase (Δmm), were compared within each group and between groups by the non-parametric Kruskal–Wallis test with *post-hoc* one-tailed Dunn’s test. Whole-blood IFN-*γ* responses to PPDB and DIGRA-C, measured as ΔOD or S/P ratio, were compared within each group by one-tailed non-parametric Mann–Whitney test. Whole-blood IFN-γ responses were also compared between vaccination groups by the non-parametric Kruskal–Wallis test with *post-hoc* one-tailed Dunn’s test. The antibody IgG responses between week 14 and week 16 were compared within each group by two-way ANOVA with matched values and *post-hoc* one-tailed Bonferroni’s multiple comparisons test. The two non-vaccinated animals were used as negative controls and were not included in the statistical analysis. GraphPad Prism version 8.0.0 software (San Diego, CA, United States) was used for the statistical analysis.

## 3 Results

No animals presented remarkable clinical symptoms or adverse reactions during the entire experiment. Non-vaccinated animals were negative for all skin tests and IGRAs, as well as for the IgG ELISA (data not shown).

### 3.1 Defined antigens enabled null or reduced vaccine-induced diagnostic interferences

All animals of the BCG i.n. and prime-boosted groups were positive to the SIT, either to low or high cutoff values, and two and one animal of HIMB and HIMBmuc i.n. groups, respectively, were positive using the low cutoff point, although only one animal of the HIMB i.n. group remained positive when using the high threshold (Table 1). Similarly, all BCG i.n. and HIMBpar-HIMBmuc vaccinated animals, and four BCG-HIMBmuc animals were positive to the CIT with low cutoff value, decreasing to 4, 3, and 3, respectively, when using the high threshold. The positivity cutoff criteria used in the SIT were also applied for the interpretation of the skin tests using the DST-F. In contrast to the SIT results, all animals were negative to the DST-F (high cutoff value) except one of the HIMBpar-HIMBmuc group. All animals of the BCG i.n., HIMB i.n., and HIMBmuc groups remained negative to the DST-F skin test when the low cutoff point was used, while three animals of the HIMBpar-HIMBmuc and one of the BCG-HIMBmuc groups became positive, respectively.

**Table 1:** Immunodiagnostic tests results.

	Skin tests						IGRAs^1^			
	Low cutoff values			High cutoff values			ID Screen® Ruminant IFNg		BOVIGAM™ TB	
Group (*n*)	SIT^2^	CIT^3^	DST-F^4^	SIT	CIT	DST-F	PPD^5^	DIGRA-C^6^	PPD	DIGRA-C
NV (2)	0^7^	0	0	0	0	0	0	0	0	0
BCG i.n (6).	6	6	0	6	4	0	5	0	3	0
HIMB i.n (6).	2	2	0	1	1	0	1	0	0	0
HIMBmuc i.n (6).	1	0	0	0	0	0	0	0	0	0
HIMBpar s.c. - HIMBmuc i.n (6).	6	6	3	6	3	1	6	4	4	1
BCG s.c. - HIMBmuc i.n (6).	6	4	1	6	3	0	6	0	4	0

^1^Interferon-γ release assay; ^2^Single intradermal tuberculin test; ^3^Single intradermal comparative tuberculin test; ^4^Fusion protein containing ESAT-6-CFP10 and EspC antigens; ^5^*M. bovis* and *M. avium* purified protein derivatives; ^6^antigenic cocktail (ESAT-6, CFP10, and EspC).

Regarding the IGRA results obtained using the ID Screen Ruminant IFNg kit, five out of six animals in the BCG i.n., all animals in the prime-boosted groups and only one animal in the HIMB i.n. group were positive using PPDs, whereas all animals, except four of the HIMBpar-HIMBmuc group, were negative when using the DIGRA-C as stimuli. Similarly, the BOVIGAM TB kit detected three positive animals in the BCG i.n. group, and four in each prime-boosted group using PPDs, whereas only one animal in the HIMBpar-HIMBmuc group was positive when using DIGRA-C.

### 3.2 Intranasal BCG induced similar cell-mediated immune responses to PPDs than parenteral regimes

**Figure 2:**
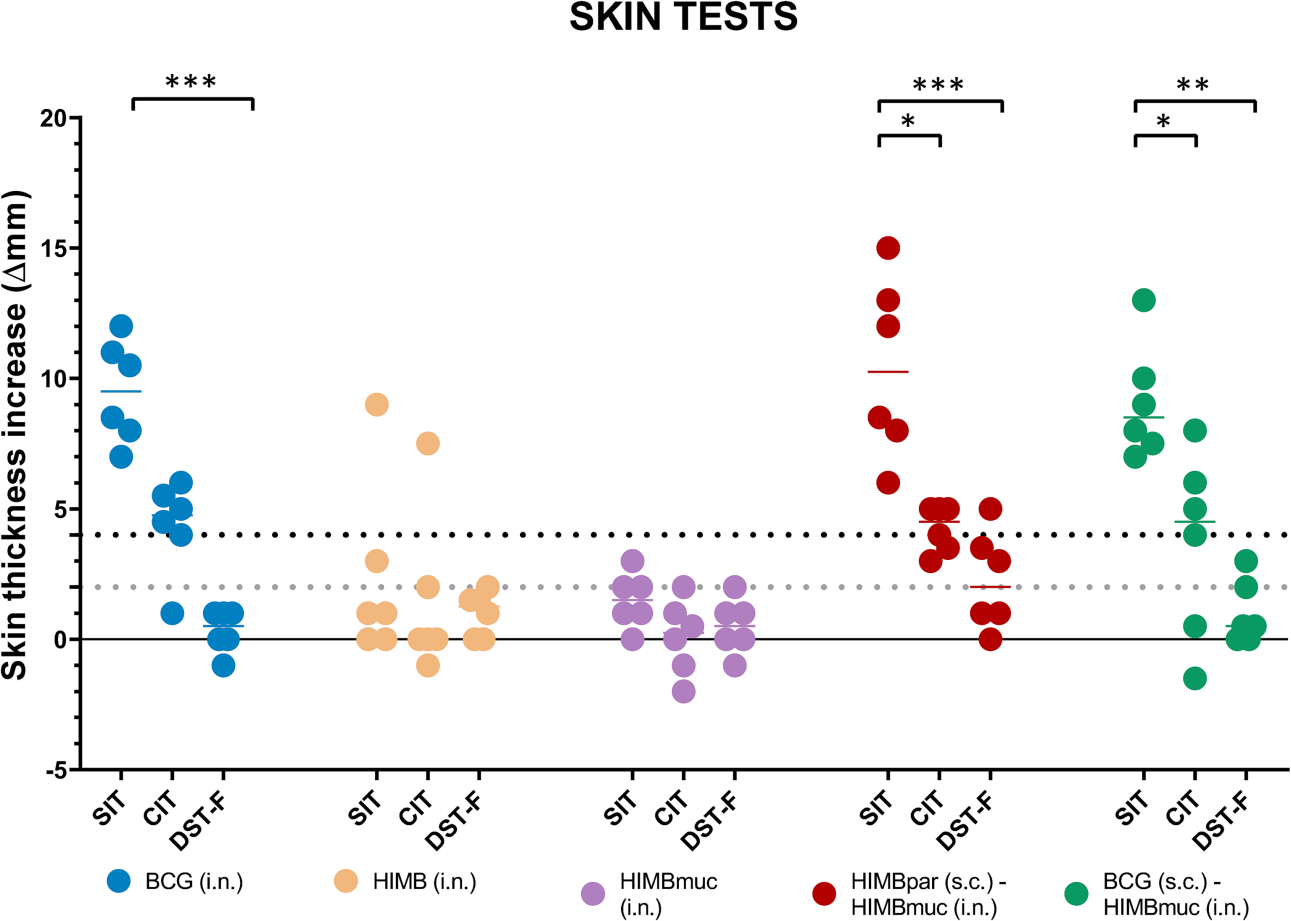
Skin fold thickness increases after PPDB, PPDA, and DST-F inoculation. PPDB: *M. bovis* tuberculin; PPDA: *M. avium* tuberculin; DST-F: fusion protein containing ESAT-6, CFP10, and EspC antigens; SIT: single intradermal cervical tuberculin test; CIT: single intradermal comparative cervical tuberculin test. Each color represents a different vaccination group. Horizontal lines in each group represent the median values. As appropriate cutoffs for DST-F have not been evaluated in goats, the interpretation of the PPDs-based assay results was done following the indications of VISAVET and adapted to the interpretation of the DST-F-based assays. The dotted lines show the positivity cutoffs for the low (gray, > 2 mm) and high (black, ≥ 4 mm) cutoff values of the SIT and DST-F skin tests. *p ≤ 0.05, **p ≤ 0.01, ***p ≤ 0.001, non-parametric Kruskal–Wallis test with *post-hoc* one-tailed Dunn’s test.

The delayed-hypersensitivity (DTH) reaction measured by the skin tests showed significantly higher responses using PPDB compared to DST-F in the BCG i.n. (*p* ≤ 0.001), HIMBpar-HIMBmuc (*p* ≤ 0.001), and BCG-HIMBmuc (*p* ≤ 0.01) groups but not in the HIMB i.n. and HIMBmuc groups (Figure 2). There were no statistically significant differences in the DTH to PPDB between the BCG i.n and both prime-boosted groups. However, PPDB-specific skin thickness increase was significantly higher in the groups BCG i.n. and HIMBpar-HIMBmuc compared to the HIMB i.n. and HIMBmuc groups (*p* ≤ 0.05). This DTH response was also higher in the BCG-HIMBmuc group compared to the intranasal HIMB groups but yet not statistically significant. The DTH showed no differences between groups when using DST-F.

**Figure 3:**
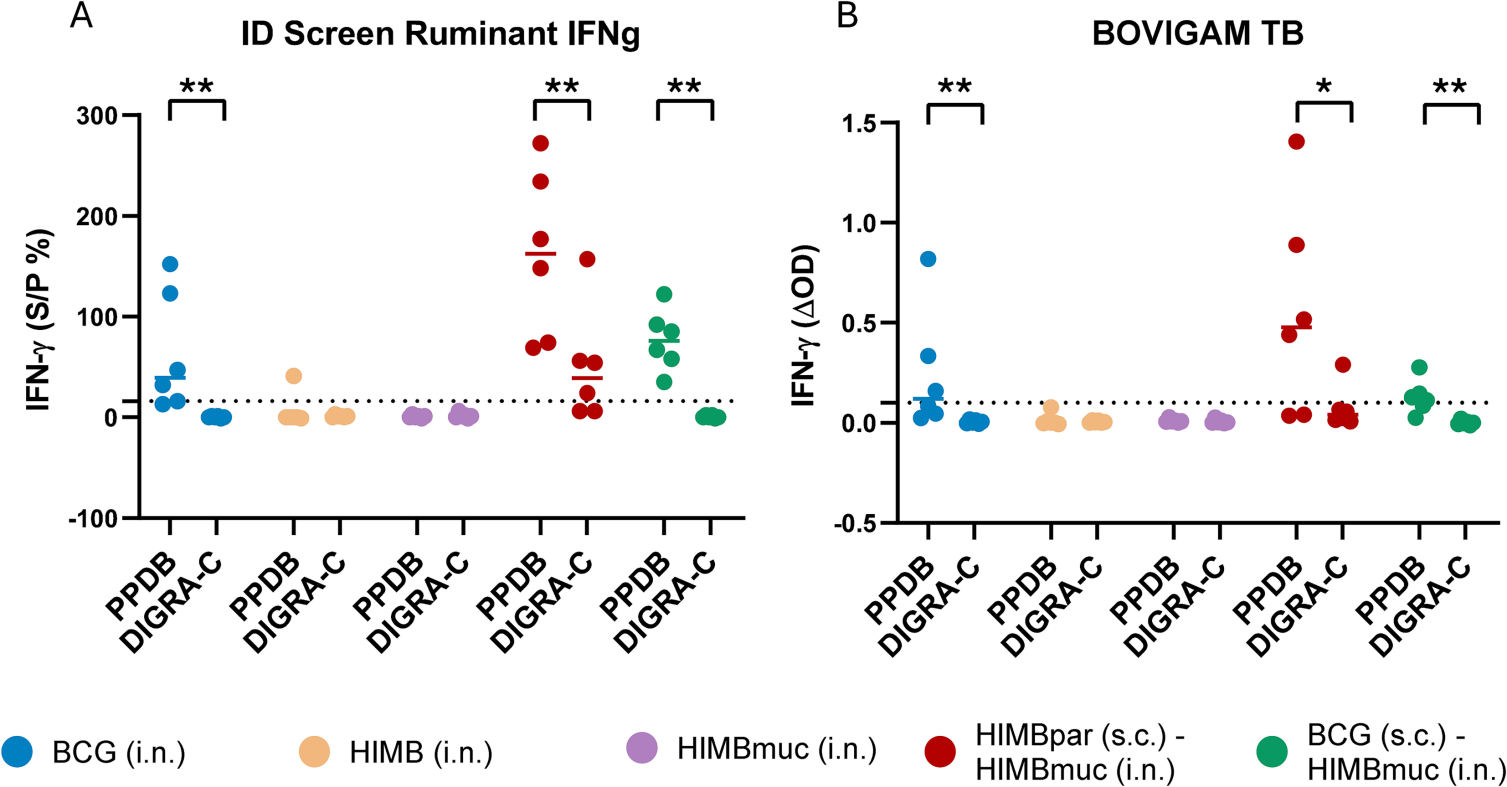
IFN-γ responses using each IGRA kit. **(A)** Antigen-specific IFN-γ levels measured with the ID Screen® Ruminant IFNg kit (ID, Grabels, France). The S/P (%) ratio was calculated by dividing the optical density (OD) PPDB minus PPDA or PBS by OD positive control-negative control x 100. Cutoff for positivity: S/*P* ≥ 16%. **(B)** BOVIGAM™ TB (Thermo Fisher Scientific, Waltham, MA, United States). The ΔOD was calculated using PPDB OD or DIGRA-C OD minus PBS OD. The dotted lines show the cutoff for positivity: ΔOD ≥ 0.1 and PPDB OD > PDDA OD for the tuberculin-based assay and ΔOD ≥ 0.1 for the DIGRA-C-based test. The interpretation of the PPDs-based assay results was done following the indications of the Spanish National Reference Laboratory for bovine TB (LCSA, Santa Fe, Granada, Spain) and adapted to the interpretation of the DIGRA-C-based assays. Horizontal lines in each group in **(A)** and **(B)** represent the median values. One-tailed non-parametric Mann–Whitney test: *p ≤ 0.05, **p ≤ 0.01.

IFN-*γ* responses were also significantly higher using PPDB compared to DIGRA-C in the BCG i.n., HIMBpar-HIMBmuc, and BCG-HIMBmuc groups using both commercial kits (Figure 3). There were no statistically significant differences in the IFN-γ PPDB-specific responses among these three groups. On the contrary, IFN-γ DIGRA-C-specific responses were significantly higher in the HIMBpar-HIMBmuc group compared to the BCG i.n. and BCG-HIMBmuc groups (*p* ≤ 0.01), when using the ID Ruminant IFNg kit, and only the BCG-HIMBmuc (*p* ≤ 0.05) group, when using the BOVIGAM TB kit.

### 3.3 HIMB prime-boosted goats showed strong antibody responses regardless of the skin test boosting effect

**Figure 4:**
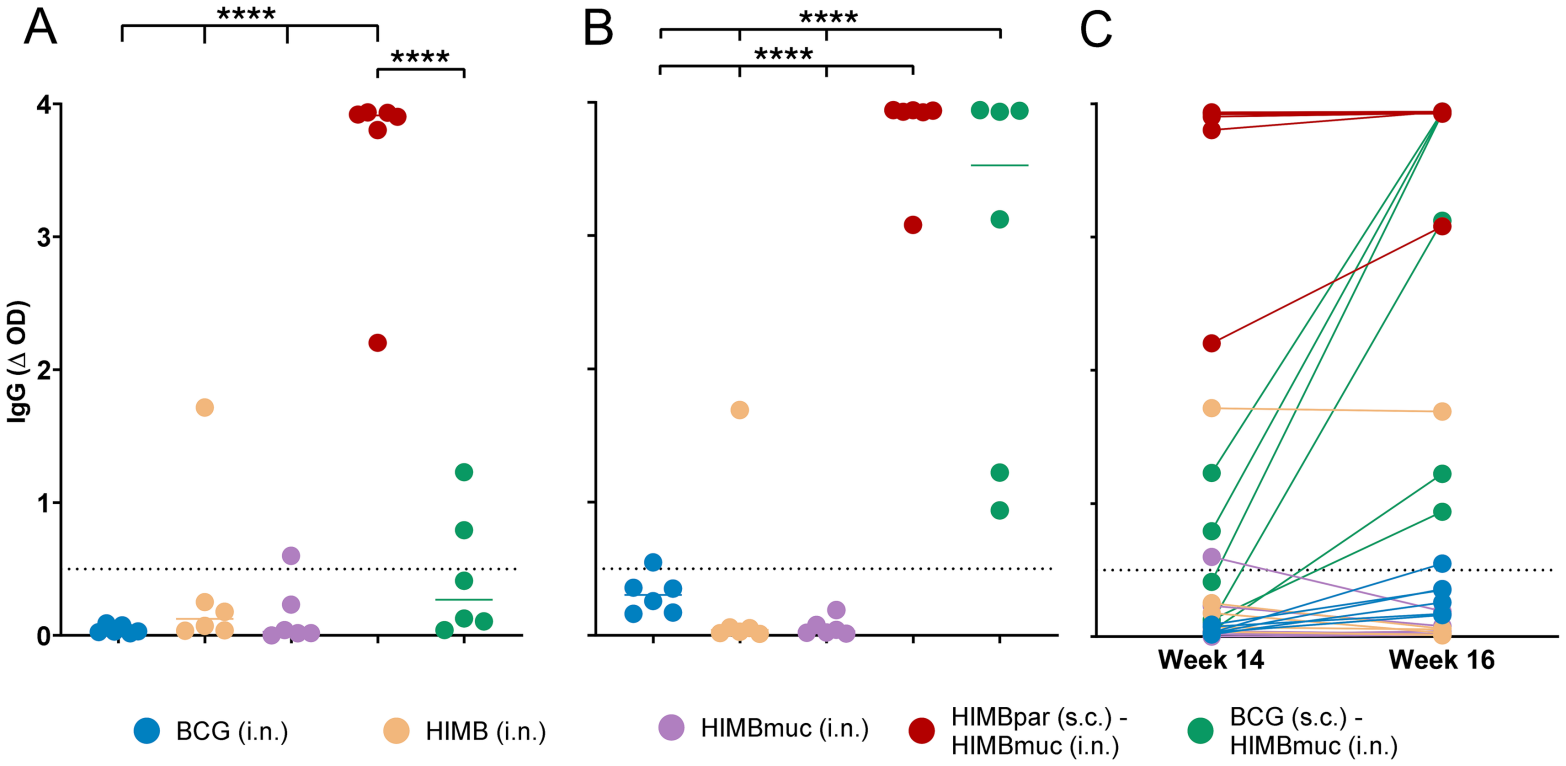
Antibody IgG responses against the MPB83 antigen. **(A,B)** IgG responses to the MTBC-specific MPB83 antigen at weeks 14 **(A)** and 16 **(B)** after s.c. vaccinations (weeks 8 and 10 after i.n. vaccinations). **(C)** Comparison between IgG plasma levels at weeks 14 and 16 within individuals. MPB83-IgG levels were calculated as OD of antigen-coated well minus OD of non-coated well (ΔOD). Continuous horizontal lines represent the median values. The dotted line shows the cutoff for positivity: ΔOD ≥ 0.5. ****p < 0.0001, two-way ANOVA with matched values and *post-hoc* one-tailed Bonferroni’s multiple comparisons test.

The MPB83-specific IgG titers of the HIMBpar-HIMBmuc prime-boosted group were significantly higher compared to the other groups at week 14 (before the skin testing, *p* ≤ 0.0001, Figure 4A). In addition, all animals of the HIMBpar-HIMBmuc prime-boosted group were seropositive, while none of the BCG i.n. animals and only one animal of HIMB i.n and HIMBmuc i.n. and two animals of the BCG-HIMBmuc groups were seropositive at that time point.

Two weeks after the skin testing performed at week 14, enhanced antibody responses were observed in the BCG-HIMBmuc group, being significantly higher compared to all the single-dose vaccinated groups (*p* ≤ 0.0001; Figures 4B,C), and all the animals of this group had already seroconverted at week 16. To a lesser extent, the skin testing also enhanced the IgG levels of BCG i.n. vaccinated animals (although they were not yet statistically significantly higher compared to the other single-dose vaccinated groups), and only one out of the six animals of this group seroconverted at week 16.

## 4 Discussion

The findings of the present study further strengthen the evidence that the use of the defined antigens ESAT-6, CFP10, and EspC can significantly reduce or even eliminate vaccine-induced interference in diagnostic tests. Across all vaccination strategies tested, both in skin tests and IGRAs, these antigens displayed minimal or null cross-reactivity. These reagents have already been successfully evaluated to distinguish between parenterally BCG-vaccinated from infected goats (23, 24). In addition, our recent studies have recently suggested that heat-inactivated mycobacterial vaccines induce minimal or undetectable specific immune responses against ESAT-6 and CFP10 in goats, these antigens being suitable to specifically identify infected animals and avoid cross-reactivity with vaccinated ones (9, 19). Since these proteins are only secreted by replicating *bacilli*, they can no longer be secreted after bacterial inactivation (18).

However, despite all goats vaccinated intranasally with single-dose vaccines showed negative results on DIVA tests, the homologous prime-boost strategy (priming with HIMBpar s.c. followed by boosting with HIMBmuc i.n.) showed false-positive animals to all DIVA tests when high cutoff points were used, mainly with the ID Screen Ruminant IFNg kit (four out of six animals were positive). This result disagrees with the absence of positivity to this test in goats parentally vaccinated with a single dose of HIMBpar (9, 19). Notably, in these studies, the EspC (Rv3615c) protein was not added in the diagnostic reagents, and diagnostic tests were performed up to 7 weeks of vaccination (instead of 14), and these differences may account for the observed variations in the results. In addition, it is also possible that a minimal content of the remaining defined antigens in HIMB vaccines would be sufficient to induce detectable immune responses when these are enhanced by the boosting effect of HIMB i.n.

On the other hand, only one animal in the heterologous BCG-HIMB i.n. prime-boost immunization schedule presented a single diagnostic interference when applying the more stringent interpretation of the DST-F skin test, whereas all animals remained negative when using high cutoff points. In addition, two animals from the HIMB i.n. and HIMBmuc groups, respectively, showed small responses to the DST-F skin test but did not yet reach the cutoff value for positivity. Therefore, the limited interference observed in the BCG-HIMBmuc prime-boost schedule may be explained by the intranasal administration of the HIMB vaccine since the expression or secretion of the defined antigens is restricted in BCG due to the lack of the Esx-1 secretion system (25).

To date, the majority of studies investigating TB vaccination routes have concentrated on subcutaneous (26), intramuscular (27), and, to a lesser extent, intravenous (28) and oral administration (29). However, the intranasal route has been relatively underexplored in the field of TB vaccination, but it has been attracting growing interest in recent times (30, 31).

Given that inhalation is the main route of MTBC entry, causing primarily respiratory disease, it has been hypothesized that vaccination via the respiratory mucosal route could improve efficacy against pulmonary TB compared to the systemic route (30). In this study, we assessed three vaccines formulated for intranasal administration alone or in combination with well-defined parenteral vaccines.

As expected from previous studies, vaccination schedules that included parenteral vaccination were highly effective in inducing a strong systemic immune response to PPDs (19, 26, 32, 33), but interestingly, we observed that a single-dose intranasal BCG vaccination induced similar DTH and IFN-*γ* responses to PPDs to those observed in goats vaccinated parenterally with BCG or HIMB. In light of these results, intranasal nebulization of BCG not only completely avoided diagnostic interference when using defined antigens but also induced robust cell-mediated systemic immune responses, representing a promising alternative to parenteral administration.

In contrast, when a single intranasal dose of HIMB was administered, either alone or in combination with an adjuvant, it induced only mild or undetectable cell-mediated immune responses using the diagnostic tools and reagents applied in this study, except for one goat of the HIMB i.n. group that was positive to SIT, CIT, and one of the two IGRAs. These results align with studies on oral vaccination using HIMB, delivered as edible bait, which suggests that while this approach can provide partial protection to vaccinated individuals, it does not elicit a strong systemic immune response (34). This suggests that the HIMB vaccine, when delivered intranasally, does not provoke the same level of systemic immune activation as either the parenteral vaccines or the BCG i.n. vaccine, but further studies are needed to conduct a more comprehensive characterization of vaccine immunogenicity and its translation to the degree of protection.

In this study, the interpretation of the DST-F-based assay results was adapted from the established interpretation for tuberculin use in goats. However, defined antigen reagents such as DST-F might not elicit immune responses in the same way as PPDs. Applying the conventional PPD criteria to these new reagents could potentially affect the interpretation of TB diagnostic results as they may not fully capture the immune responses associated with defined antigens. On the other hand, using the threshold tailored to cattle reactivity to DST-F (response considered positive if *Δ* skin thickness ≥ 2 mm (14)) can influence the accuracy and specificity of the TB diagnosis in goats since one more animal of each of the groups HIMB i.n., HIMBmuc, and BCG-HIMBmuc would be positive when using the cattle cutoff compared to the adapted tuberculin cutoff for goats. Establishing specific reactivity criteria for defined antigen reagents could improve the diagnostic reliability of these new tools in the goat species.

When comparing the two IGRA kits used in this study, the ID Screen Ruminant IFNg kit showed slightly higher vaccine-induced interferences on a tuberculin-based test when compared to the BOVIGAM TB kit, in terms of both higher PPDB-specific IFN-*γ* levels and a greater number of individuals being classified as positive for TB. Notwithstanding, the two kits use different approaches for classifying positive and negative animals, and it may also have consequences on the diagnostic performance (35).

Finally, significant differences between treatment groups were also found in serology. Previous research has described that both infected cattle (36) and goats (24) experienced a boosting effect of MTBC-specific antibody responses 2–3 weeks after skin testing. In contrast, HIMB has been shown to induce strong antibody responses without the need for this boosting effect from skin tests (9, 19, 37), unlike BCG (19). Consistent with this, in our study, all animals of the HIMBpar-HIMBmuc group seroconverted prior to skin testing, and IgG titters in this group were significantly higher compared to the other vaccination groups at that time point. On the contrary, both HIMBmuc and HIMB i.n. did not elicit significant antibody titters in plasma, and seroconversion was only detected in one animal from each group, indicating that the intranasal route of administration may not be as effective in inducing systemic antibody responses compared to the parenteral one.

Two weeks after the skin test, antibody responses in the BCG-HIMBmuc group were elevated to levels comparable to those observed in the HIMBpar-HIMBmuc group. This suggests a lack of induction by BCG of early detectable MPB83-specific humoral responses compared to HIMB until these are enhanced through skin testing. It could be a consequence of the retained expression of MPB83 protein in BCG strains compared to wild-type *M. bovis* (38). Furthermore, no seroconversion was observed in any animal that received intranasal BCG prior to skin testing, after which a mild increase in antibody levels was observed, resulting in only one seropositive animal with IgG titers just above the threshold.

Interestingly, the same animal of the HIMB i.n. group that was positive for IGRA and skin tests also exhibited significantly stronger humoral responses compared to the other animals in the group, suggesting a more pronounced immune activation that set it apart from its counterparts. Moreover, one animal in the HIMBmuc group shifted from a weak seropositive status at week 14 to seronegative by week 16, just after undergoing the skin test. We hypothesize that this fluctuation may indicate a non-specific response that is not directly associated with the administered vaccine.

## 5 Conclusion

Among the different vaccination regimes evaluated in this study, the heterologous primer-boost strategy with BCG s.c. followed by HIMBmuc i.n. and single-dose intranasal administration of BCG demonstrated higher diagnostic specificity in DIVA skin test and IGRA compared to the homologous prime-boost strategy (HIMB s.c. followed by HIMBmuc i.n.) while exhibiting similar systemic cellular immune responses to PPDB. Based on these results, further research could focus on efficacy studies to evaluate the potential of these two vaccination strategies in the protection against the MTBC challenge. The results also reinforce the suitability of combining the defined antigens ESAT-6, CFP10, and EspC to improve the accuracy of TB diagnostics in vaccination settings.

## Data Availability

The raw data supporting the conclusions of this article will be made available by the authors, without undue reservation.
